# Resistance to (Digital) Change: Individual, Systemic and Learning-Related Perspectives

**DOI:** 10.1007/978-3-030-55878-9_13

**Published:** 2020-07-15

**Authors:** Antonia B. Scholkmann

**Affiliations:** 1 Learning, Design and Technology / UNESCO Deputy Chair of Data Science in Higher Education Learning and Teaching, University of Mannheim / Curtin University, Mannheim / Perth, Germany; 2 Department of Education and Social Sciences, University of Cologne, Köln, Germany; 3 Learning, Design & Technology, University of Mannheim, Mannheim, Germany; 4 Human Sciences Faculty, University of Cologne, Cologne, Germany; Aalborg University, Aalborg, Denmark

**Keywords:** Resistance to change, Employee resistance to change, Resistance to digital transformation, Field theory, Learning theory, Knut Illeris

## Abstract

Resistance to change has been elaborated on from different perspectives: with a focus on employee resistance to change and as a systemic phenomenon, but also in the light of digital change and digital transformation. However, an integration of these approaches is not easy to find. This chapter discusses the phenomenon of resistance to change in light of current understandings of the concept as well as new elaborations, which might help to pinpoint specific challenges of digital change resistance. To this end, I will dive into the research traditions that have been built up around the concept. In order to understand resistance to digital change, specifically, I will draw upon the theory of Danish educational researcher Knut Illeris and explore the potential of his writings to explain resistance to digital change from a learning perspective. Throughout I will use examples from higher education digitalization research, to illustrate the respective phenomena. Key navigation points of this chapter are to elaborate resistance to (digital) change both as an individual and a systemic phenomenon and to contribute to a better understanding of resistance to digital change in light of incremental and disruptive change expectations.

## Introduction: Resistance to Change – A Topic for the Digital Sphere?

In the wake of the far-reaching changes that digital transformation of society and work brings about, it is worthwhile also exploring the meaning of resistance to change. Digital transformation can be seen as an almost unprecedented change – both for human nature and for the nature, content, and organization of learning and work (Nagy and Koles 2014; Matzler et al. 2018; Meyer 2010; Peres et al. 2019). Because of the magnitude, the phenomenon of resistance to change also needs to be integrated into the picture. However, we must not make the mistake of applying the concept only mechanistically, or with a short-sighted lens to only some aspects such as individuals allegedly “resisting” change. Instead, a broad understanding is needed – both of the overall phenomenon of change and what it does with the individuals involved in it.

Resistance to change can, without doubt, be labeled as one of the big buzzwords in the organizational development literature, and searches on databases such as Google Scholar or ResearchGate provide impressive numbers of contributions with “resistance to change” and “employee resistance to change” as search terms. The undoubted popularity and huge scientific interest in the phenomenon can on the one hand be interpreted as the overall extent of the problem – being that it seems incredibly common that resistance to (organizational) change happens (Bareil 2013). Also, the academic writings around this concept are filled with accounts about failed or dried-up change initiatives being attributed to various forms of resistance, both from employees and stakeholders (e.g., Battistelli et al. 2013; Kuroda et al. 2016; Nov and Ye 2009; Oreg 2003; Röth and Spieth 2019; Self 2007; among others). On the other hand, though, the scientific concept of resistance to change has been critiqued as a simplification of more complex and multifaceted processes, which play out in the wake of (organizational) change, which in itself is a complex and by no means fully explained phenomenon (Dent and Goldberg 1999).

Resistance to change has also been elaborated on with respect to digital change. In general, it can be said that change in the digital sphere is, at least conceptually, talked about as a highly disruptive enterprise, with a focus on fundamental transformations of both practices and products (e.g., Loonam et al. 2018). However, when it comes to resistance to change, the researched construct is often technology implementation, and the researched “resistances” were actually related to rejection of certain (new) technologies (e.g., Laumer 2016). And although a good number of theorists have argued that implementation and consequent adoption of new technologies is the first step towards more fundamental digital transformations (e.g., Berghaus and Back 2017; Matt et al. 2015; Murdoch and Fichter 2017; Vial 2019), the notion that digital transformation must always be disruptive has also been challenged by some (e.g., Furr and Shipilov 2019). Either way, when looking at resistance in the context of digital change, the question of whether this resistance is against an incremental implementation of new technology or a disruptive shift of an organization’s identity is yet uncharted.

In this chapter, I will discuss the phenomenon of resistance to change in the light of current understandings of the concept as well as on some new elaborations, which might help to pinpoint specific challenges of digital change resistance. For this, I will dive into the research traditions that have been built up around the concept and especially into two research strands, which conceptualize resistance to change either as a phenomenon related to the individual, i.e., *employee resistance to change*, or as a *systemic phenomenon*. In order to understand resistance to digital change specifically, I will then draw upon the theory of Danish educational researcher Knut Illeris and explore the potential of his writings to explain resistance to digital change under a learning perspective. Throughout, I will use examples from my own field of expertise, specifically the digital transformation in higher education, to illustrate the respective phenomena. Key navigation points of this chapter are to elaborate resistance to (digital) change both as an individual and a systemic phenomenon, and to contribute to a better understanding of resistance to digital change in the light of incremental and disruptive change expectations.

## Change and Resistance

### Change and Resistance – Current Understandings

As can be seen from the examples from the digital field, change can mean a lot of different things: the implementation of new technology to digitalize a previously analogue process, or the complete disruptive transformation of a whole business model, its products, customers, and values (e.g., Hinings et al. 2018). These different notions relate to long-known theoretical concepts of different modes of organizational learning , such as single- versus double-loop learning (Argyris and Schön 1978) or the exploration-exploitation dichotomy (March 1991). In variations, these theories postulate that change can either be radical and transformative or incremental, either directed towards new and innovative solutions or towards the refinement of proven solutions.

Change, it is said proverbially, is the one constant in the world. However, learning and organizational change literature is divided over the question of whether change is so natural that we all are constantly changing, and very readily so, or is an exception, which will “disturb” the normal business only temporarily (Tsoukas and Chia 2002). From a systemic perspective, it can be said that systems on the one hand crave stability and internal balance, which makes them per default resistant to change. However, since the world is developing dynamically, systems need to outbalance themselves towards this and create equilibrium by change. As has been argued, this creates the paradoxical situation that, although a system craves stability, it also needs functioning mechanisms to manage change in order to adapt efficiently to changing conditions (Burnes 2015; Boxenbaum and Pedersen 2009).

As a tangible concept, resistance to change tends traditionally to be defined along the lines of any behavior of individuals or groups which oppose managerial decisions (for an overview, cf. Burnes 2015), that is, as “(…) active or passive responses on the part of a person or group that militate against a particular change, a program of changes, or change in general.” (Peiperl 2005, p. 348). Although this definition mentions both active and passive responses, the term “militate” is somewhat misleading here, since it might imply overt and hostile behaviors. However, as can be inferred from the second part of this definition, resistance can also show as defiance, nonbehavior, or other forms of passiveness towards the envisioned change (for an overview on active and passive change-resistance indicators, cf. also Piderit 2000).

Psychologically, resistance has been defined as happening on the behavioral, the cognitive, or the emotional level, or, of course, through a combination of these (Piderit 2000). But when it comes to defining concrete and observable indicators, the literature is somewhat ambiguous as to what should be counted as “resistance.” Some studies have tied to operationalize resistance, for example, as inflexibility towards job changes (McGuinness and Cronin 2016); others have pointed out emotional reaction or cynicism (Grama and Todericiu 2016). A common notion throughout the literature also seems to be that resistance will show in the failure of an envisioned change imitative and resulting losses in productivity and revenue (e.g., Matt et al. 2015) – thus “blaming the less powerful for unsatisfactory results of change efforts” (Krantz 1999, p. 42). Newer research has engaged in reframing resistance as ambiguous feelings towards change (e.g., Piderit 2000).

### Employee Resistance to Organizational Change

In the current literature , the phenomenon of resistance to change is discussed with a strong focus on employee resistance to organizational change. Under the assumption of people’s “natural tendency to prefer keeping to what is well-known and familiar rather than to accept innovation, and thus the unknown” (Laumer 2016, p. 1), many publications are concerned with unravelling causes for these individual resistances, and with formulating management advice on how they can be overcome. Part of this literature thereby focuses on resistance as a personality trait or disposition (e.g., Nov and Ye 2009; Oreg 2003), which makes certain individuals more prone to resist change than others. The majority of publications, though, finds causes for resistance in values, motives, emotions, cognitive structures, and cultural norms of individuals, that play together to make said individuals hesitant or overtly hostile towards an intended change in their organization (e.g., Danışman 2010; Howard and Mozeiko 2015; Jost 2015; Pardo del Val and Martínez Fuentes 2003; Oreg 2006). Measures to overcome this employee-related resistance to change are also located at different levels: work-psychological measures, such as increased task autonomy or feedback, are promoted (Battistelli et al. 2013); organizational development in a broader sense is advocated for sense making (Röth and Spieth 2019) or the integration of several facets of resistance (Cervone 2011; O’Connor 1993), while humanistic recommendations favor, for example, the concept of spirituality (Lawton 2017).

While parts of the respective literature show a tendency to position individuals as potentially “defiant” entities in the otherwise unproblematic change process, this notion of resistance to change that neglects the interpersonal nature of the phenomenon has been challenged. In a more “modern” (Bareil 2013) interpretation, employee’s resistance of change is treated as important feedback about potential flaws within a change process, and mismatches among employees’ motives, needs, and values and those brought about by the intended change (Harvey and Broyles 2010; Perren 1996).

What is problematic with both perspectives – the traditional and the modern alike – is the fact that they allocate the phenomenon unilaterally on the side of the change recipients, who are seen as either sabotaging the process or serving as the “canary in the coalmine” to optimize it. Under specific critique stands the relation between change agents, i.e., actors who, on behalf of the organization, promote the change, and change recipients, i.e., actors who are the carriers of the change measures. Here, it has been argued that a focus on employees as change recipients as the (sole) location of resistances neglects the dynamics between the different stakeholder groups (i.e., change agents) involved (e.g., Ford et al. 2008; Klonek et al. 2014; cf. also 2.2). Also, it has been shown, based on the study of acceptance and resistance towards policy-induced changes in hospitals, that the dichotomy between change agents and change recipients can be artificial, since these social roles can and will change dynamically over time during the process (McDermott et al. 2013). The same study also challenges the dichotomy between acceptance and resistance to change by showing the variety of reactions towards this kind of mandated changes. Last but not least, the construction of resistance to change as employee resistance to organizational change bears an inherent power imbalance, since this endows the change agent with the unilateral capacity to diagnose resistance and the power to overcome it (Thomas and Hardy 2011; Vos and Rupert 2018).

### Resistance to Change as a Systemic Phenomenon

Already more than 20 years ago, Dent and Goldberg (1999) pointed out that the oversized focus on employees as the major force of resistance to change might have originated in a misunderstanding of the original conceptualization of the term by German-American organizational researcher Kurt Levin (cf. also Ford et al. 2008; Mathews and Linski 2016; McDermott et al. 2013): In his field theory, Lewin described the phenomenon of resistance towards organizational change as arising either from a lack of strong enough forces to induce change, or from the prevalence of too strong barriers towards these forces that hinder the occurrence of change in a given system (Lewin 1947). From a systemic perspective, it can be said that systems on the one hand crave stability and internal balance (*homeostasis*), which makes them per default resistant to change. However, since the world is developing dynamically, systems need to outbalance themselves towards this and create (new) equilibrium by change (Goldstein 1988). Given that, change will happen if either the external pressures on the system are strong enough to disturb its homeostasis, or if the system’s barriers towards the outside are weak or low enough for new information to break through. Taking this perspective, resistance must be seen as the phenomenon of a stall to change that can be caused by a multitude of influences, under which the individual is only one factor (Kotter 1995).

The idea that resistance to change emerges from a complex interplay between driving and resisting forces can be found in studies which focus on the dynamics of change. For example, it has been shown that during change-related communications, change-recipients show information about the prevalence of driving versus blocking change forces, and that change agents can provoke these forces in the respective communications (Klonek et al. 2014). Also, it has been pointed out that a systemic view allows the analysis of institutions and organizations as being resisting entities to change: for example, when they object to the (legitimate) demands of minorities and marginalized groups to reduce discriminations (Agócs 1997). To address the complex interplays between driving and resisting forces, Lewin’s theoretical groundwork has been extended towards the concept of action research (Burnes 2004), which serves as an organizational development approach to dynamically integrate resistance – also in the wake of digital transformation (Argyris 1993; Baskerville and Myers 2004; Chevalier and Buckles 2019).

The idea of competing field forces can eventually also be extended to understand broader dynamics of change and resistance, and this view resonates well with research on digital change in the field of higher education. Here it has been researched and discussed for many years that the mere implementation of learning technology has not led to substantial transformations in teaching practices (e.g., Blin and Munro 2008; Kirkup and Kirkwood 2005). For the last two decades, various forms of “resistance” on the teachers’ side have been discussed, as have more complex explanations (e.g., Torrisi-Steele and Drew 2013; Matrosova Khalil 2013). With the arrival of the Covid-19 pandemic and the closing down of physical education in many countries, the state of digital transformation of higher education has changed dramatically. Put bluntly, all “resisting” barriers to digital teaching at this point seem to be outweighed by a steep increase in the power of the forces demanding (immediate) change (Kerres 2020). It still needs to be studied, however, to what extent this ad hoc change will lead to sustainably transformed digitalized practices beyond crisis mode.

## Addressing Resistance to Digital Change as a Learning Challenge

… when it comes to digital transformation, *digital* is not the answer. *Transformation* is. (Westermann 2018, p. 116)

As elaborated in the first paragraphs of this chapter, digital change is often expected to yield huge transformative and disruptive powers (Jesse 2018; Matzler et al. 2018). Given that digital change is in its core about transformation, the individual and its resistances comes back into focus. However, the role of the individual here is not that of an opposing force, as it tends to be conceptualized in research on employee resistance to change. Instead, the individual here can be seen as an “agent of change” (Syakdiyah et al. 2019, p. 165), who acts as the mediating entity between macrolevel organizational changes and microlevel enacted behaviors (Schmid 2019). In this notion, engaging with or “resisting” change becomes a question of engaging in or resisting learning.

Although, generally speaking, “(t)he relationship between individual and organizational learning remains one of the contested issues in organizational learning debates” (Antonacopoulou 2006, p. 455), it is the understanding that individuals and their learnings form the foundations of change at the group and organizational level (e.g., Kim 1998). Also, analogies have been drawn between the (psychological) research on learning processes and the development and change of organizations (Cohen 1991; Döös et al. 2015; Rodan 2008). Interestingly, theoretical and empirical underpinnings for the conceptualization of the individual as the actual carrier of organizational change and transformation can be explicitly found in writings from the sphere of digital change, again, where an interplay between conceptual changes within the individual’s cognitive structures and consequent transformations in organizational identity is being proposed (e.g., Jahn and Kurse 2019; Murdoch and Fichter 2017).

### Knut Illeris: Dimensions, Processes, and Types of Learning

In order to grasp this idea further, I want to dive into the learning theory of Knut Illeris (2003, 2009a, b, c, 2017), which I propose as a comprehensive framework by which to understand how individual learning dynamics can be related to organizational change. In his theory, which can be seen as a synthesis of other theorists’ works (cf. Illeris 2009a, p. 8), the author conceptualizes three dimensions and two processes of learning. Within the individual, learning takes place as a balancing of the dimensions of *content* (knowledge, understanding, skills) and *incentives* (motivation, emotion, volition). These internal dimensions are supplemented with a third, which is the *interaction* (action, communication, and cooperation) between the individual and its environment. Not unequal to the previously described force field assumptions of Lewin, learning is triggered also in this theory by the interaction between the individual’s internal regulation processes and the affordances of the external world. (For a more detailed overview, cf. Illeris 2003, 2009a, b, c.)

According to Illeris’ assumptions, learning will happen in different forms (or “types”, Illeris 2009a, b, c, p. 8): as *cumulative learning* , being a simple add-on procedure to stock up factual knowledge; as *assimilative learning*, being the integrating of new information into existing mental schemes; as *accommodative* *learning*, being the adaption of mental schemes to fit with new information; or as a new type of learning, labeled either *significant, expansive, transitional,* or *transformative learning* (based on the respective theorists, cf. Rogers and Freiberg 1994; Engeström 2015; Alheit 1994; Mezirow 1991), which comprehensively means an extensive rearrangement of mental schemes and human identities (Illeris 2015).

### “Nonlearning” as Resistance to (Digital) Change

Illeris’ writings provide a systematization of so-called nonlearning phenomena, which theoretically can be used to interpret resistance to change. The first is *mislearning*, which is related to the content dimension and describes instances where content other than the intended is learned, either by accident or lack of attention. Also, mislearning can only be clearly detected with relatively simple tasks where a clear detection of “wrong” content is possible (Illeris 2017, pp. 158). The second phenomenon is *defense against learning*, which, related to the incentive dimension, describes the “classical” motivational resistance. Defense against learning is assumed to happen mostly subconsciously and is therefore seen as hard to address. It can show in various subforms such as open rejection, blocking, distortion , or neurotic symptoms (ibd., pp. 160). The third phenomenon is *resistance to learning*, related to the interaction-dimension. As opposed to defense, resistance to learning is active and conscious, thus energizing, which makes this reaction also a potential basis for the initiation of transformative learning experiences (Illeris 2009a, p. 16).

**Fig. 13.1:**
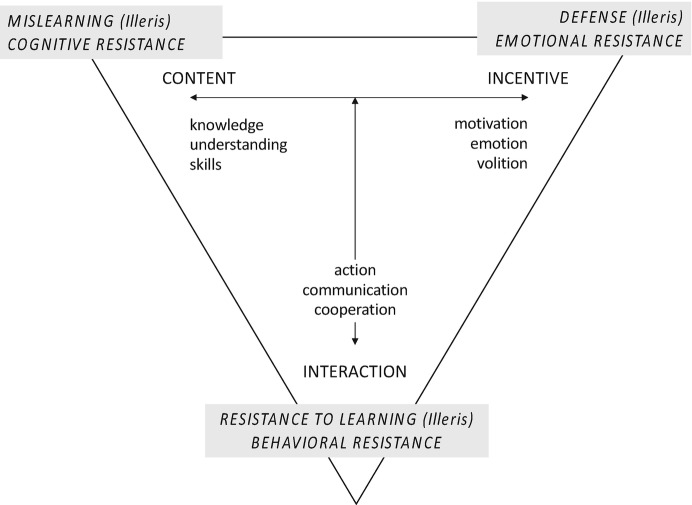
Dimensions of learning, nonlearning, and resistances to change. (Based on Illeris 2009a, b, c)

As might be already obvious, these three types of nonlearning resonate with the conceptualizations of employee resistance to change in Sect. 13.2.1, and, more generally, with the three proposed psychological dimensions of resistance, being emotional, cognitive, and behavioral (cf. Piderit 2000) (cf. Fig. 13.1). Mislearning, in a way, can be seen as the equivalent to cognitive resistance, which here should be seen as an intended or unintended (mis-)interpretation of the change content. Applied to the envisioned transformative learning under digital change initiatives, mislearning can, for example, mean misunderstandings about the nature of technologies, their functionalities, etc., and a resulting failure to use and/or transform them.

Defense against learning shows similarities with emotional resistances, which makes sense especially since both concepts have been described as being rooted in early understandings of subconscious rejections based on internal psychodynamics (Burnes 2007; Illeris 2009a, b, c). With that, defenses/emotional reactions are certainly the hardest category to address, since they (can) relate to more or less deeply rooted emotional experiences and are often not consciously accessible. At the same time, they are also the closest to the “target dimension” of transformative (digital) change – that is, to a person’s identity, and therefore can provoke substantial resistance.

Resistance to learning, described as an active process, mirrors the behavior-related dimensions of resistance to change, and in both Illeris’ theory and in the change management literature, the behavior dimension is seen as the point of entry to work towards developing creative solutions (Austin and Bartunek 2003; Illeris 2009a). However, neither the literature on resistance to change nor Illeris are very outspoken about possible forms this resistance can take. For the time being, we can assume that, related to the desired transformative learning taking place, resistances here might show as active oppositions – for example, by questioning proposed measures – which can serve as starting points for integrative and transformative conversations (e.g., Matthews 2019).

### Mismatches in Learning – An Undetected Form of “Resistance”?

As elaborated in the previous paragraph, the nonlearning dimensions in Illeris’ theory can be used to describe and systematize individual resistances in specific situations of (digital) change processes. However, they are clearly rooted in the individual, and thus lag behind from a more systemic perspective, which is able to describe perceived resistances to change as an interplay between individual and organizational forces and barriers. As an addition to the elaborations made before, I would like to use another part of the theory to broaden the perspective on how resistance to digital change can eventually be interpreted in this sense. This part relates to the four types of learning mentioned above.

Applied to the sphere of digital transformation, we can assume that individuals will potentially engage with technology in these four modes:

- *Cumulative:* Learning about technology, i.e., acquiring basic knowledge about tools and their functionalities
- *Assimilative:* Learning how to use technology to perform well-known procedures
- *Accumulative:* Learning to do new procedures based on the opportunities of new technology
- *Transformative-expansive:* Generating new ideas to understand, structure, and influence the world

These modes of engagement can happen based on these individuals’ assumptions what a given situation of change calls for, i.e., given on their interpretation of this situation (Illeris 2015). Perceived “resistance” to change in this light can be interpreted as a mismatch between an expected learning activity and executed learning activity.

This assumption might sound technical in nature: by ensuring an adequate match between the desired form of organizational change (being either incremental or disruptive) and the corresponding learning activity by the individual, leaders and managers should be able to design resistance-free change processes. However, as discussed earlier, change, and resistance towards it, must be understood as a complex interplay between organizational and individual driving and blocking forces, and it may not be possible to engineer resistance-free processes to a perfect degree. The idea of matching must be interpreted more widely in this case: as a negotiated and communicated mutual understanding of what a specific situation calls for to ensure successful change, and which forms of learning might match with organizational learning and change needs (Augustsson et al. 2013; Boateng 2011; Ji Hoon Song and Chermack 2008; March and Olsen 1975).

It also needs to be stressed, again, that conceptualizing an alleged resistance to change as a mismatch between expected and enacted learning practices does not touch on the other forms of resistance to learning (cognitive and emotional) from the model above. While these other forms allow for their allocation within the individual, the mismatch-conceptualization addresses the interplay between individual and organizational learning. Illeris (2004) himself has elaborated on how the interaction-dimension bridges into collective learning processes in the workplace, where the individual’s learning potentials and practices interact with the technical-organizational and the social-cultural work environment and constitute an enacted work practice (cf. also Illeris 2011).

To underpin this idea, I am drawing again on evidence from the sphere of digital transformations of higher education. Programmatic writings here have advocated the potential of digital change for far-reaching transformation and changes, both in terms of the extent and innovation of technology use, but also at the level of the underlying pedagogical assumptions and practices (e.g., Duignan 2020; Meyer 2010; Salmon 2014). However, a broad corpus of studies has shown that the actual practices, mainly in the arena of digital teaching and learning, lag behind on transformative or even accumulative practices (e.g., Blin and Munro 2008; Lai and Hong 2015). This effect, it can be hypothesized, can be attributed to the fact that signals within the respective institutions or systems encourage prioritizing cumulative or assimilative approaches to technology (Hinings et al. 2018). For example, it has been argued that many institutional digitalization strategies in higher education prioritize the digitization of teaching material over a change in digitalized teaching practices (Sandkuhl and Lehmann 2017), although others, where in place, have been shown to lead to higher technology integration rates compared to where no institutionalized strategy has been in place, overall (Tømte et al. 2019).

Also, as has been analyzed with respect to the evolvement of higher education learning management systems, these tended to be used following “traditional” conceptions about teaching and learning, resulting in an assimilative usage of these to follow transmissive learning conceptions (Van den Berk 2013). In line with that, it could also be shown that digital transformation in some areas of research, which also have advanced practices of knowledge sharing and networked collaborations, is far more advanced than it is in higher education teaching (Scanlon 2014). Last but not least, higher education teachers themselves have expressed that in order to use digital technologies in more advanced (accommodative or transformative) ways, they do not need more specific technological support but crave helpful relationships (e.g., by academic developers) to scaffold their transformative changes at the crossroads between technology and pedagogical identity (e.g., Ching and Wittstock 2019; Thoring et al. 2018). Accordingly, it could also be shown that practices which led towards a shared collective understanding and institutionalization of digitally enhanced teaching and learning held the potential for overcoming barriers in adoption (Martins and Baptista Nunes 2016).

## Resistance to Digital Change – Unanswered Questions

This chapter presented a suggestion on how to disentangle the phenomena of resistance to change and digital transformation, and to explore their interwoven and common grounds. To this end, I elaborated in the first part of this chapter on current understandings of both change and resistance as concepts in organizational development research, and on possible implications of this for understanding the phenomena of resistances to (digital) change. In the second part, I presented and discussed the learning theory of Knut Illeris as a comprehensive approach to understand resistance to digital change – and especially transformative change. However, as the phenomenon of digital transformation is a topic “in the making,” so too are my elaborations. Naturally, this leaves open ends at this point, and some topics need to be explored further.

Framed as a question, we firstly need to ask what organizations can do to create a climate which outbalances driving and resisting forces to (digital) change, constructively, and in which adequate change is enabled through matching expectations and executions of learning. As elaborated in the beginning of this chapter, the concept of resistance to change shows a tendency to circle around the individual as both the source of and the solution to this phenomenon. Even in the present text this is prevalent, since its focus is on individual learning as the basis of organizational change. Taking seriously that resistance to change is in fact multifaceted and systemic, we can assume that the organization acts as an autonomous entity in this, which holds valid interests that need to be mediated through leadership (e.g., Amy 2008). Processes of negotiation and coconstruction can clarify change objectives, goals, and practices, which allow for the creation of shared understanding of learning needs and directions at the organizational and individual level As with other fields of organizational theory and research before, the field of (higher) education can provide an interesting template here since it already holds high degrees of self-organization and collegial negotiation practices, which should be used and bridged towards digital transformative processes (Scholkmann 2011; Vial 2019).

A second question is in how far digital transformation can be an imposed, mandated change process, after all – given that identity transformations are at its core? As research from the tradition of Scandinavian New Institutionalism (Boxenbaum and Pedersen 2009) on the implementation of managerial concepts has shown, a mere top-down transfer will likely lead to the resistance form of “ceremonial” adoptions, with no change in practices or identities (Sahlin and Wedlin 2008). Also, some authors have argued, again for the field of higher education, that a “collective willingness to change” (Graf-Schlattmann et al. 2020, p. 19) is needed to overcome field-specific resistances and to bring about sustainable digital change. An important point here seems to be that the adoption and transformation of a concept cannot be expected to result in solutions that look equally similar. Instead, variations must be seen as legitimate local reinterpretation of an idea (c.f. Scholkmann 2020, for an example on how this applies to educational change), which stresses again the need for collective and interactive interventions which lead to transformed digital practices and identities both at the individual and at the organizational level.

A third question, moreover, is in which ways resistance to changes relates to the concept of agile organizations, and whether agility can be seen as way to overcome the sometimes “traditional” notions of resistance, especially with the focus on employee resistance and the dichotomy of change agents and change recipients. At least one study has explored resistances and barriers that were prevalent in the wake of an agile digital transformation project (Nerurkar and Das 2017), while another has dived into the creative potential of crises that can happen in a digitally transformed company (Kazanjian et al. 2000). However, more research and theory seem to be needed to better understand the dynamics of change acceptance and resistance that can arise in new forms of work beyond traditional hierarchical institutions.

Last but not least, it can be asked whether the comparison of change and learning applied in this chapter stands up to close scrutiny of these two concepts. The notion of change and learning following the same principles has been argued by learning researchers engaged in inquiry-based and problem-based learning and by organizational development researchers, alike (Chidiac 2013; Loyens et al. 2015). Also, learning and development theories seem to underpin these assumptions (e.g., Vygotskij and Cole 1981). However, a more thorough exploration and empirical underpinning of these assumptions could be a worthwhile enterprise.
